# Effect of Baseline Characteristics and Tumor Burden on Vaspin Expression and Progressive Disease in Operable Colorectal Cancer

**DOI:** 10.3390/diagnostics10100801

**Published:** 2020-10-09

**Authors:** Jung-Yu Kan, Yi-Chen Lee, Yu-Da Lin, Wan-Yi Ho, Sin-Hua Moi

**Affiliations:** 1 Division of Breast Surgery, Kaohsiung Medical University Hospital, Kaohsiung 80756, Taiwan; kan890043@gmail.com; 2 Department of Surgery, Kaohsiung Medical University Hospital, Kaohsiung 80756, Taiwan; 3 Department of Anatomy, School of Medicine, College of Medicine, Kaohsiung Medical University, Kaohsiung 80708, Taiwan; yichen83@kmu.edu.tw (Y.-C.L.); wayehe@kmu.edu.tw (W.-Y.H.); 4 Department of Electronic Engineering, National Kaohsiung University of Science and Technology, Kaohsiung 807, Taiwan; yudalinemail@gmail.com; 5 Center of Cancer Program Development, E-Da Cancer Hospital, I-Shou University, Kaohsiung 82445, Taiwan

**Keywords:** colorectal cancer, vaspin, progressive disease, partial least squares path modeling, tumor burden

## Abstract

Colorectal cancer is a highly heterogeneous malignancy in the Asian population, and it is considered an important prognostic factor for baseline characteristics, tumor burden, and tumor markers. This study investigated the effect of baseline characteristics and tumor burden on tumor marker expression and progressive disease in colorectal cancer by using partial least squares variance-based path modeling (PLS-PM). PLS-PM can be used to evaluate the complex relationship between prognostic variables and progressive disease status with a small sample of measurements and structural models. A total of 89 tissue samples of colorectal cancer were analyzed. Our results suggested that the expression of visceral adipose tissue-derived serpin (vaspin) is a potential indicator of colorectal cancer progression and may be affected by baseline characteristics such as age, sex, body mass index, and diabetes mellitus. Moreover, according to the characteristics of tumor burden, the expression of vaspin was generally higher in each progressive disease patient. The overall findings suggest that vaspin is a potential indicator of the progressive disease and may be affected by the baseline characteristics of patients.

## 1. Introduction

Colorectal cancer is a highly heterogeneous malignant disease with a high prevalence in the Asian population; in addition to causing a poor quality of life, it accounts for high cancer-related morbidity and mortality due to its poor prognosis, especially in those with disease progressed lesions [1,2,3,4,5]. Studies have classified the prominent clinicopathological factors into multiple categories based on their features [6,7]. Clinicopathological factors including the baseline characteristics, tumor burden, and tumor markers, which can be affected or associated with short- and long-term survival outcomes in colorectal cancer, are considered to be prognostic factors [8,9,10]. Baseline characteristics of patients with cancer include the age at which cancer was diagnosed, sex, and comorbidity status. Generally, factors that are directly measured from the tumor lesion are considered tumor burden factors. In addition to indicating the molecular expression from cancer cells in the tumor, tumor markers play a definite role in cancer diagnosis. The different clinicopathological factors that are usually associated with each other interactively affect the prognostic outcome [11].

Vaspin, a visceral adipose tissue-derived serine protease inhibitor, is associated with a specific mechanism in cancer [12,13]. Generally, vaspin expression is associated with obesity, body mass index (BMI), and insulin sensitivity [14,15,16]. A study reported a potential role for vaspin in carcinogenesis and its promising role as a biomarker in cancer development [13]. Consistent findings have demonstrated the role of vaspin as an adipocytokine that could affect colorectal cancer progression [17]. However, few clinical studies have reported the effect of tumor burden on vaspin expression and cancer prognosis; moreover, the related evidence in colorectal cancer is lacking.

Generally, the partial least squares variance-based path modeling (PLS-PM) approach is used to evaluate the causal–predictive relationship in a study or a highly complex hypothesis with low theoretical evidence [18]. Unlike traditional parametric statistical approaches, PLS-PM is less restrictive for a small sample size and enables the examination of latent variables by using measurement and structural models [19]. For instance, the clinicopathological factors involved in the estimation of progressive disease can be associated with various latent variables according to the characteristics including the baseline characteristics, tumor burden, and tumor markers. Thus, the effect of each latent variable on progressive disease status can be measured using the measurement and structural models of PLS-PM.

Studies have particularly reported the effect of the patient’s baseline characteristics, tumor burden, and molecular markers on progressive disease in cancer [8,9,10]. However, the effect of baseline characteristics and tumor burden on the expression of tumor markers (such as vaspin) and progressive disease has seldom been discussed. Moreover, the complex causality in cancer is usually difficult to estimate using parametric analysis with limited samples. Hence, this study investigated the effect of baseline characteristics and tumor burden on tumor marker expression and progressive disease outcomes by using a path modeling approach.

## 2. Materials and Methods

### 2.1. Data Sets

Eighty-nine colorectal cancer tissue samples were collected from newly diagnosed patients following surgical treatment between January 2006 and December 2007. Data sets were retrospectively collected from the Department of Surgery of Kaohsiung Medical University Hospital under the protocol approved by the institutional review board (KMUH-IRB-E(I)-20160035; approved on 24 March 2016). Retrospective cancer prognosis variables included age at which cancer was diagnosed, sex, BMI, diabetes mellitus (DM), histological grade, pathological stage, tumor size, lymph node status, perineurial invasion (PNI), vascular invasion (VNI), Carcinoembryonic Antigen (CEA), and vaspin expression. Progressive disease in cancer was considered as the main prognostic outcome. Each patient was followed up regularly to monitor cancer progression status, and in the study patients, progressive disease was histologically confirmed; conversely, disease-free participants were indicated as those with no progressive disease during the study period. All participants were followed-up from the first date of the diagnosis of cancer to the first date of diagnosis of progressive disease or until the end of the study. The data of patients lost to follow-up before the end of study were censored.

### 2.2. Immunohistochemistry of Vaspin Expression

Immunohistochemistry staining for vaspin was performed on an automated Bond-Max autostainer (Leica Microsystems, Bannockburn, IL, USA). Slides with tissue sections from paraffin-embedded tissue blocks were deparaffinized at 72 °C and rehydrated in Bond Wash Solution. Heat-induced epitope retrieval was conducted using Bond Epitope Retrieval Solution for 20 min at 100 °C, followed by peroxide block placement for 5 min at room temperature. The slides were incubated with vaspin (1:50 dilution, ABGENT, San Diego, CA, USA) antibody for 30 min at room temperature. Antibody detection was performed using 3,3′-diaminobenzidine tetrahydrochloride for 1 min at room temperature. The slides were counterstained with hematoxylin, followed by mounting and examination using light microscopy. A negative control was obtained by substituting the primary antibody with the immunoglobulin fraction of nonimmune rabbit serum for each staining (Supplementary Figure S1). All images were captured using a Nikon E-800M microscope (Tokyo, Japan) and then processed using PhotoImpact X3 (Ottawa, Canada). The score for vaspin expression was determined by calculating the percentage of cytoplasmic staining (from 0% to 100%). Only staining in tumor cells (approximately 500 cells in 4–6 high-power fields) confirmed histologically as adenocarcinomas (arising in enterocytes and goblet cells of glandular epithelium) were calculated. Two pathologists with no prior knowledge of the patient’s clinical information performed the immunostaining of vaspin of each specimen separately; however, in the rare occasion of a discordant score, the specimens were re-evaluated and scored based on a consensus.

### 2.3. Statistical Analysis

The prognosis-associated variables were grouped according to cancer progressive disease status. Distribution of the included variables was appropriately summarized using frequency (percentage), median (interquartile range), or mean (standard deviation [SD]), and the difference between disease-free and progressive disease patients was appropriately estimated using the χ² test, Fisher’s exact test, or independent two-sample t test. The PLS-PM method was used to evaluate simultaneous relationships among the included prognosis-associated variables and progressive disease in cancer. PLS-PM enabled the examination of the complex relationship between prognostic variables and progressive disease status through both measurement and structural models without any sample size restriction [19]. In this study, we divided the prognostic variables and progressive disease status into four latent variables: baseline characteristics (BASE: age, sex, BMI, and DM), tumor burden (BUR: grade, stage, tumor size, node status, PNI, and VNI), tumor molecular status (MOL: CEA and vaspin expression), and progressive disease status (metastases, META). The expression (percentage) of vaspin according to different tumor characteristics was illustrated using a boxplot, and the difference in vaspin expression according to the disease-free status in each subgroup was estimated using the Mann–Whitney test. Data were analyzed using the R and plspm package [20].

## 3. Results

The prognostic variables of colorectal cancer according to progressive disease status are summarized in Table 1. A significantly higher proportion of patients with progressive disease had advanced stages (stages III–IV vs. I–II: 76.5% vs. 32.7%, *p* < 0.001), greater tumor size (T3–4 vs. T1–2: 67.3% vs. 100%, *p* < 0.001), lymph node invasion (N1–2 vs. N0: 67.6% vs. 32.7%, *p* = 0.001), vascular invasion (VNI, yes vs. no: 67.6% vs. 36.4%, *p* = 0.004), and perineural invasion (PNI, yes vs. no: 79.4% vs. 50.9%, *p* = 0.007). In addition, patients with progressive disease demonstrated significantly higher expression of both carcinoembryonic antigen (CEA, *p* = 0.003) and vaspin (*p* = 0.021). The median level of CEA in patients with progressive disease was 11.3 (1.7-1228), whereas that in disease-free patients was relatively low at 3.5 (0.7–110). Similarly, the mean level of vaspin expression in patients with progressive disease was 40.1 ± 23.1, whereas that in disease-free patients was 27.4 ± 26.0.

The factor loadings and cross-loadings in the measurement model are given in Table 2. The bold fonts indicate the factor loadings of each variable on their respective latent variable, and others indicate the cross-loadings of each variable on distinct latent variables. For instance, the factor loadings of CEA and vaspin expression in MOL were 0.742 and 0.774, respectively. However, the cross-loadings of CEA and vaspin in META were 0.312 and 0.244, respectively. The communality index indicates the quality of the measurement model for each latent variable, and the redundancy index indicates the quality of the structural model for each latent variable; a higher redundancy index represents better quality. The measurement model results indicated that vaspin expression had the highest redundancy index and a communality index of 0.600 (approximate to 0.7), suggesting reasonable quality of the measurement results.

**Figure 1:**
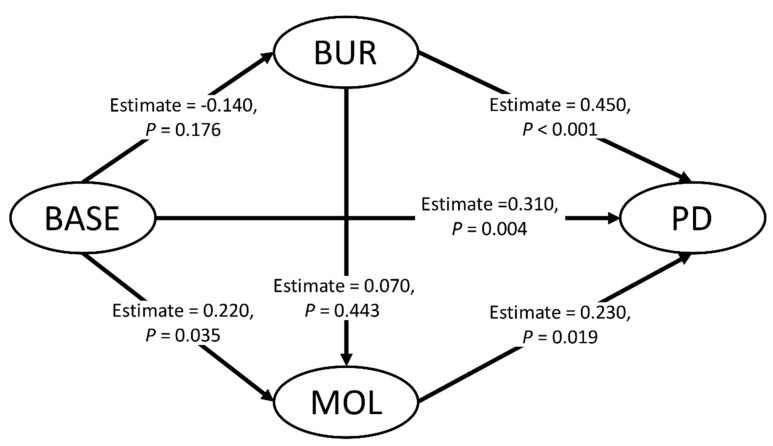
Structural model. BASE: baseline characteristics, including age, sex, BMI, and DM; BUR: tumor burden, including grade, stage, tumor size, node status, PNI and VCI; MOL: tumor molecular status, including CEA and vaspin expression; META: progressive disease status.

Figure 1 presents the structural model results. All beta path coefficients were positive, except for that for the relationship between BASE and BUR. BASE was nonsignificant and negatively correlated with BUR with a weak coefficient estimated (estimate = −0.14, *p* = 0.176), whereas BASE showed a significantly positive relationship with MOL with an estimated coefficient of 0.22 (*p* = 0.035). BUR and MOL had a nonsignificant positive relationship (estimate = 0.07, *p* = 0.443). Conversely, META had a significantly positive relationship with BASE (estimate = 0.31, *p* = 0.004), BUR (estimate = 0.45, *p* < 0.004), and MOL (estimate = 0.23, *p* = 0.019).

**Figure 2:**
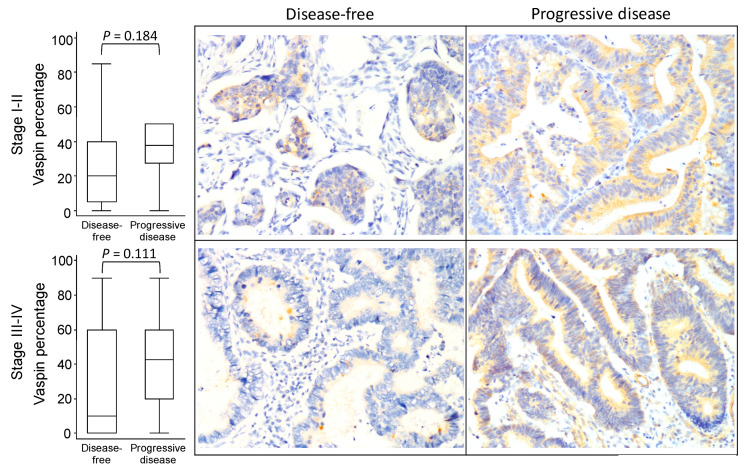
Vaspin expression boxplot and immunostaining imaging results in different tumor stage according to progressive disease status in colorectal cancer.

**Figure 3:**
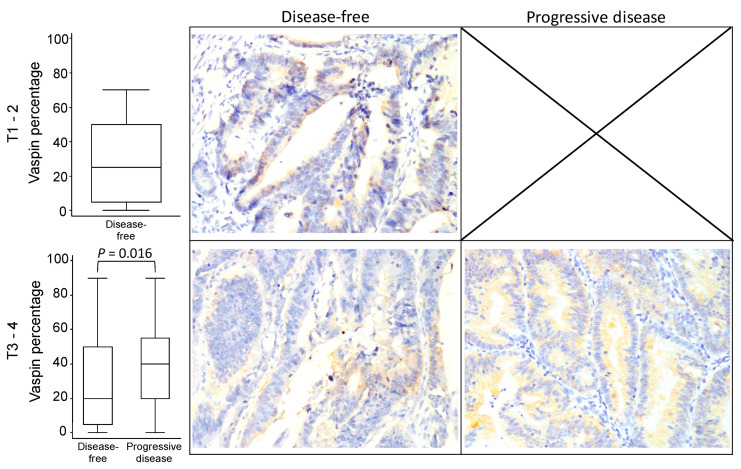
Vaspin expression boxplot and immunostaining imaging results in different tumor sizes according to progressive disease status in colorectal cancer. X or the blank box indicates no sample within the category.

**Figure 4:**
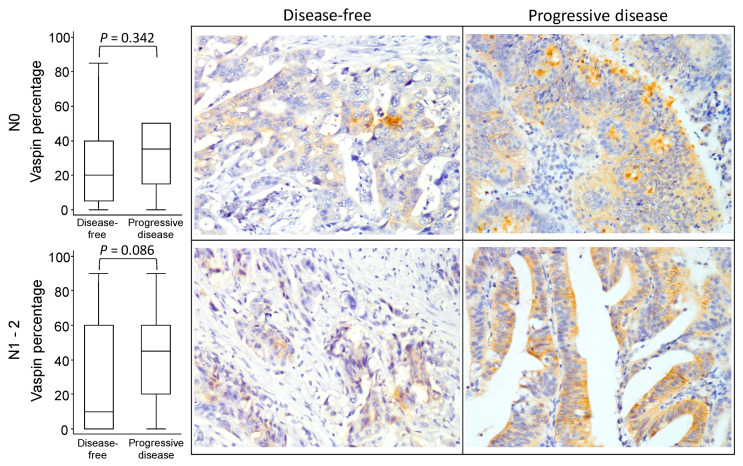
Vaspin expression boxplot and immunostaining imaging results in different lymph node invasion status according to progressive disease status in colorectal cancer.

Figure 2, Figure 3 and Figure 4 illustrates the vaspin immunostaining imaging results in the tumor burden subgroups according to the progressive disease status. Although the boxplots indicate patients with progressive disease in the subgroups of tumor stage (Figure 2), tumor size (Figure 3), and lymph node invasion status (Figure 4) were more likely to exhibit high expression of vaspin, a significant difference in vaspin expression between disease-free and progressive disease participants was found only in the T1 subgroup (*p* = 0.016). The results demonstrated that patients with progressive disease with different tumor burden characteristics generally had higher vaspin expression.

## 4. Discussion

The major contribution of this study is that it reported the effect of baseline characteristics and tumor burden on tumor markers according to the progressive disease status in colorectal cancer status of colorectal cancer using the path modeling approach. The study results demonstrated that vaspin expression is a potential indicator of colorectal cancer progression status and might be affected by baseline characteristics including age, sex, BMI, and diabetes mellitus (DM).

Consistent with previous findings [21,22], tumor markers including CEA and vaspin can predict colorectal cancer progression. CEA is a promising tumor marker of the prognosis of colorectal cancer; a high level of CEA usually indicates a poor prognosis or survival outcome [21,22]. Unlike CEA, vaspin is a novel biomarker of the risk of the development and progression of certain cancers; however, it has been seldom discussed in progressive disease in colorectal cancer [12,23]. The current study demonstrated that vaspin and CEA may be interdependently related to colorectal cancer progression and can be affected by the baseline characteristics.

A study indicated vaspin expression as an adipocytokine, and it is considered to increase with age and be independent of sex [24]. Moreover, vaspin is associated with the lipid profile and is highly related to BMI; variations in the serum vaspin concentration are significantly associated with BMI [14,25]. Vaspin has also been reported to be associated with insulin regulation and the development of DM, and it has been noted as potential novel biomarker of the early prediction or diagnosis of certain diabetes types [26,27]. Furthermore, a clinical trial identified vaspin levels to be associated with compensatory mechanisms for high BMI and insulin resistance in elderly participants [28]. Hence, baseline characteristics might affect vaspin levels individually or synergistically.

Inflammation is known to be a hallmark of cancer and is indicated with growing evidence to be related with an increase in cancer risk [29,30]. Epidemiological and experimental studies have provided an association between inflammation, tumorigenesis, and cancer prognosis, including colorectal cancer [31,32]. It has been reported that the association between adipocytokine mediated inflammation and tumor development [17,33]. However, the exact mechanism underlying vaspin in relation to colorectal cancer and inflammation remains to be elucidated.

PLS-PM is a novel nonparametric method that can provide a model-based inference results for complex causality study [29,30,31]. This path modeling approach allows us to specify a biological inference model and, thus, evaluate the causal interpretability between the latent variables in colorectal cancer progression. Moreover, unlike the general structural equation model approach, PLS model enables a data-driven exploration of the prediction model using a limited sample size [32]. This is one of the superior advantages that allows many small clinical studies to obtain reasonable data-driven explanatory results without requiring a large sample size. Owing to the novel causality model and small sample size in the current study, the PLS-PM analysis was chosen to provide a rationale data-driven result based on our study hypothesis.

This study has some limitations that should be noted. First, the retrospective nature of the current study limited the inclusion of potential covariates reported previously [33]; in addition, genomic-level factors were not considered [34]. Second, the small sample was obtained from a single center, which also limited the generalization of the study findings. However, the application of the PLS-PM method could provide certain model-based biological inferences based on the study hypothesis. Third, the effect of time was also neglected in PLS-PM, which might affect the credibility of the study results. Despite these limitations, this study still contributes findings, including those from the investigation of the effect of baseline characteristics and tumor burden on the expression of tumor markers according to the disease-free status in colorectal cancer.

## 5. Conclusions

To the best of our knowledge, this is the first study using PLS-PM to investigate the effect of tumor burden on vaspin expression and colorectal cancer progression. The study findings indicate that the expression of vaspin can be immensely affected by the baseline characteristics of patients with colorectal cancer, and the interaction of vaspin with CEA plays a potential role in cancer progression. Moreover, the results also indicate that vaspin, generally, is more expressed in patients with metastases of all strata according to the tumor burden characteristics. Therefore, the overall findings suggested vaspin as a potential indicator of cancer progressive disease events, and vaspin expression might be affected by the patient’s baseline characteristics. However, it is necessary to use a comprehensive pathophysiological approach to elucidate the complex relationship between prognostic adipocytokines and antigens to encourage precise treatment insights for colorectal cancer.

## Figures and Tables

**Table 1:** Prognostic variable distribution according to progressive disease status in colorectal cancer patients (*n* = 89).

Variables	Characteristics	Disease-Free (*n* = 55)	Progressive Disease (*n* = 34)	*p*
Age	Age ≥ 65 years	65.8 ± 13.2	63 ± 12.7	0.324
Sex	Male: Female	34: 21	25: 9	0.256
BMI	Body mass index ≥ 24	22.8 ± 3.6	23.7 ± 4.1	0.256
DM	Diabetes mellitus	15 (27.3%)	13 (38.2%)	0.279
Grade	Grade III	6 (10.9%)	2 (5.9%)	0.345
Stage	Stage III–IV	18 (32.7%)	26 (76.5%)	<0.001
Tumor	Tumor size (T3–4)	37 (67.3%)	34 (100.0%)	<0.001
Node	Lymph node invasion (N1–2)	18 (32.7%)	23 (67.6%)	0.001
PNI	Perineurial invasion	28 (50.9%)	27 (79.4%)	0.007
VCI	Vascular invasion	20 (36.4%)	23 (67.6%)	0.004
CEA	CEA level (ng/mL)	3.5 (0.7–110)	11.3 (1.7–1228)	0.003
Vaspin	Vaspin percentage	27.4 ± 26.0	40.1 ± 23.1	0.021

**Table 2:** Factor loadings and cross-loadings in a measurement model.

	Variables	Latent Variables	BASE	BUR	MOL	META	Communality	Redundancy
1	Age	BASE	**0.750**	−0.196	0.176	-0.106	0.563	0.000
2	Sex	BASE	**0.079**	0.241	0.186	0.120	0.006	0.000
3	BMI	BASE	**0.171**	−0.068	−0.088	0.122	0.029	0.000
4	DM	BASE	**0.643**	−0.041	0.059	0.115	0.414	0.000
5	Grade	BUR	−0.088	**0.115**	0.064	−0.085	0.013	0.000
6	Stage	BUR	−0.026	**0.789**	0.248	0.425	0.622	0.013
7	Tumor	BUR	−0.139	**0.648**	0.140	0.396	0.420	0.009
8	Node	BUR	−0.042	**0.771**	0.282	0.340	0.594	0.012
9	PNI	BUR	−0.039	**0.633**	0.240	0.285	0.401	0.008
10	VCI	BUR	−0.262	**0.630**	0.032	0.304	0.396	0.008
11	CEA	MOL	0.053	0.234	**0.742**	0.312	0.550	0.067
12	Vaspin	MOL	0.207	0.183	**0.774**	0.244	0.600	0.073
13	Progressive disease	META	0.047	0.503	0.365	**1.000**	-	-

Bolded fonts indicate factor loadings. BASE: baseline characteristics, including age, sex, BMI, and DM; BUR: tumor burden, including grade, stage, tumor size, node status, PNI and VCI; MOL: tumor molecular status, including CEA and vaspin expression; META: progressive disease status.

## Supplementary Materials

Supplementary materials can be found at https://www.mdpi.com/2075-4418/10/10/801/s1.
