# Supplementary material for: Effect of Baseline Characteristics and Tumor Burden on Vaspin Expression and Progressive Disease in Operable Colorectal Cancer
Source: Diagnostics (Basel). 2020 Oct 9;10(10):801. doi: 10.3390/diagnostics10100801 (PMC7600084; doi:10.3390/diagnostics10100801)

Figure S1. The negative control of immunostaining shown no vaspin expression for specificity of the staining in colorectal cancer.

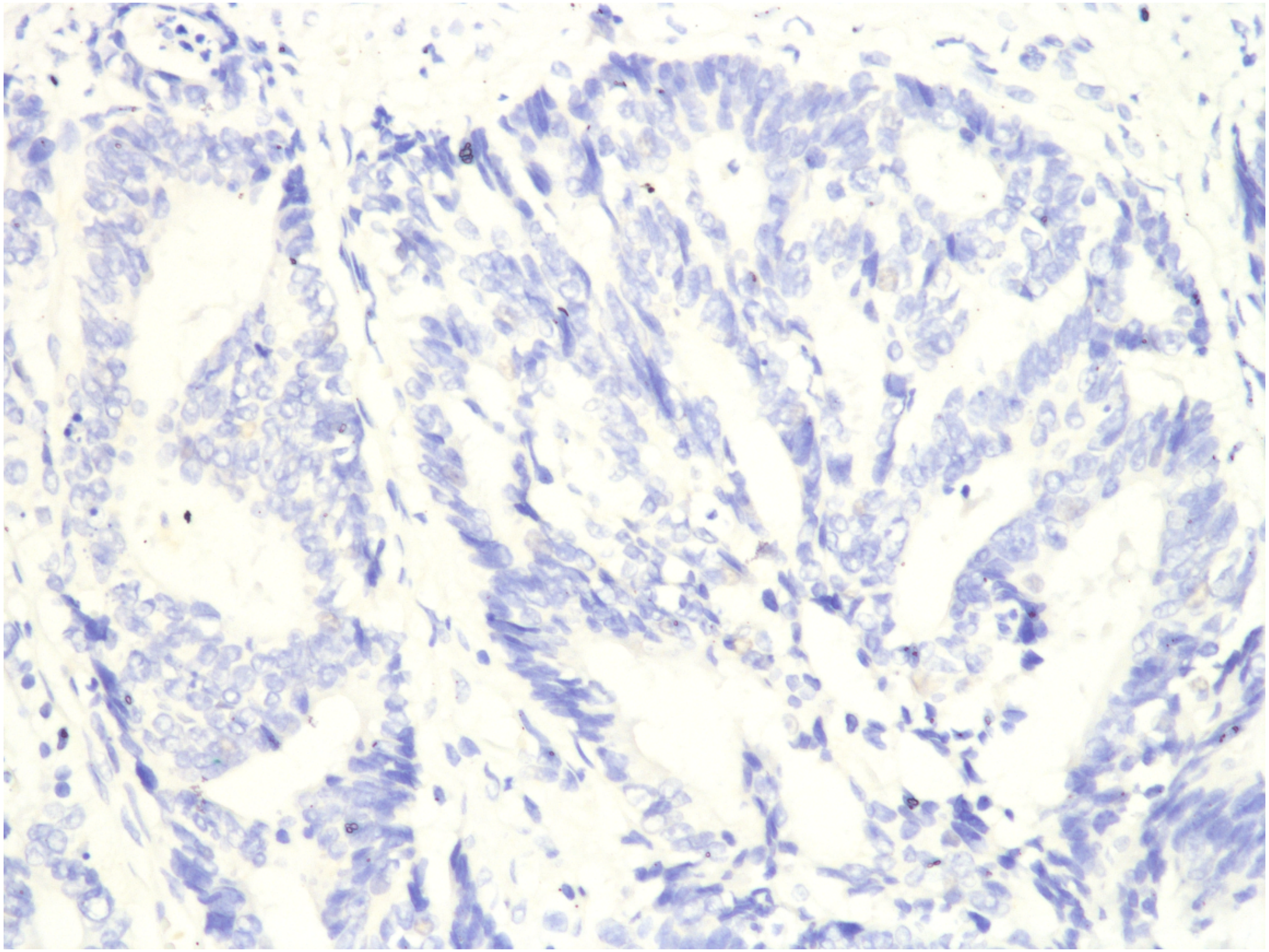

Supplement: Supplementary file 1 [file diagnostics-10-00801-s001.pdf]
